# Ethical considerations of the vaccine development process and vaccination: a scoping review

**DOI:** 10.1186/s12913-023-09237-6

**Published:** 2023-03-14

**Authors:** Habib Jalilian, Mahdi Amraei, Elnaz Javanshir, Khosro Jamebozorgi, Farzad Faraji-Khiavi

**Affiliations:** 1 Department of Health Services Management, School of Health, Ahvaz Jundishapur University of Medical Sciences, Ahvaz, Iran; 2 Cardiovascular Research Center, Tabriz University of Medical Sciences, Tabriz, Iran; 3 Faculty of Medicine, Zabol University of Medical Sciences, Zabol, Iran; 4 Social Determinants of Health Research Center, Ahvaz Jundishapur University of Medical Sciences, Ahvaz, Iran

**Keywords:** Bioethics, Beneficence, Non-maleficence, Autonomy, Medical ethics, COVID-19, Vaccination, Justice

## Abstract

**Background:**

Various vaccines have been developed and distributed worldwide to control and cope with COVID-19 disease. To ensure vaccines benefit the global community, the ethical principles of beneficence, justice, non-maleficence, and autonomy should be examined and adhered to in the process of development, distribution, and implementation. This study, therefore, aimed to examine ethical considerations of vaccine development and vaccination processes.

**Methods:**

A scoping review of the literature was conducted based on the Arkesy and O’Malley protocol to identify eligible studies published until November 2021. We searched Web of Science, PubMed, Scopus, and SciELO databases. The search was conducted using combinations of Medical Subject Heading (MeSH) search terms and keywords for Ethics, COVID-19, and vaccines in abstract, keywords, and title fields to retrieve potentially relevant publications. We included any study that reported one of the four principles of medical ethics: autonomy, justice, non-maleficence, and beneficence in the COVID-19 vaccine development and distribution and implementation of vaccinations. Letters, notes, protocols, and brief communications were excluded. In addition, we searched gray literature to include relevant studies (ProQuest database, conferences, and reports). Data were analyzed using framework analysis.

**Results:**

In total, 43 studies were included. Ethical considerations concluded two themes: (1) production and (2) distribution and vaccination. The production process consisted of 16 codes and 4 main Categories, distribution and vaccination process consisted of 12 codes and 4 main Categories. Moreover, the ethical considerations of special groups were divided into four main groups: health care workers (HCWs) (five codes), children and adolescents (five codes), the elderly (one code), and ethnic and racial minorities (three codes).

**Conclusion:**

Due to the externalities of pandemics and the public and social benefits and harms of vaccination, it is not feasible to adhere to all four principles of medical ethics simultaneously and perfectly. This issue confronts individuals and policymakers with several moral dilemmas. It seems that decision-making based on the balance between social benefit and social harm is a better criterion in this regard, and the final decision should be made based on maximizing the public benefit and minimizing the public harm.

## Introduction

The side effects of COVID-19 on public health are well known, especially if the necessary measures for social distancing are not taken to control transmission. Strict and compulsory social distancing restrictions have considerable effects on the economy, education, freedom of the people, and societies’ physical and mental health [1–3]. Therefore, the most desirable way to control the epidemic, which provides long-term protection against the outbreak of COVID-19, is to develop and distribute an effective and safe vaccine [4, 5]. With the rapid growth of vaccine research and entering the different types of vaccines into the third phase of clinical research and the issuance of emergency licenses for their global use, various ethical challenges have been raised, which are generally based on the four principles of bioethics: justice, non-maleficence, benefit, and autonomy [6]. These challenges can be classified into four general categories, including (1) safe and standard manufacturing and passing scientific and ethical stages, (2) evaluation and monitoring of safety and efficacy, (3) mass production, fair distribution, and (4) public acceptance [7]. Justice is a fair adjudication consisting of distributive justice, rights-based justice, and legal justice. Distributive justice means equal distribution of scarce resources among all; equality means the absence of avoidable or compensable disparities between different groups, countries, ethnicities and races, and societies in general [6, 8]. The principle of beneficence states that COVID-19 vaccines perform to benefit individuals and increase their well-being. Autonomy in health care gives the patient the right to make decisions regarding their health care independently based on their beliefs, attitudes, and judgments. The principle of non-maleficence or to do no harm means “not harming” the fundamental and vital principles in health care and research [8, 9]. In the past, vaccines have always been developed through a series of steps that may take years. At present, given the urgent need for COVID-19 vaccines, Human Challenge Trials (HCTs) are a potential way to accelerate the development of vaccines and treatments [10, 11]. Clinical trials raise several ethical issues, including human trials, placebo use, patent, participants’ consent, considerations for children and pregnant women, the right to post-trial access for the control group, access to affordable, safe, and effective vaccines that should be considered in the development and manufacture of COVID-19 vaccines [12].

The COVID-19 epidemic also showed that there are deep inequalities between communities and populations concerning health care, which relate to various factors, including different access to resources and socio-economic factors. After producing several COVID-19 vaccines and issuing emergency licenses for them, questions are raised about how to adhere to the ethical principle of justice and equitable distribution of vaccines among countries and even within a country to prioritize the access of different groups. These questions encompass complex and controversial issues such as economics, public perspectives/views, diplomacy, public health, and other considerations. Therefore, vaccination as an appropriate measure to prevent COVID-19 requires planning and implementation to achieve the principles of justice [13, 14]. Given the development of COVID-19 vaccines in a pandemic emergency, this can significantly affect ethical considerations in vaccine production as well as distribution and vaccination processes. So far, various studies have examined the ethical considerations of the COVID-19 vaccine, but most of them have examined ethical considerations in vaccine production or distribution and implementation of vaccination or in specific groups.

The primary objective of this study was to find out the ethical considerations related to the production, distribution, and vaccination processes. The secondary objective was realizing the ethical considerations in specific groups (i.e. children and adolescents, the elderly, health care workers, and ethnic and racial groups). The results of this study can be used as a comprehensive and integrated ethical framework for governments, international organizations, and stakeholders related to the development, distribution, and implementation of vaccination. Moreover, applying these results can lead to improving the safety and efficacy of vaccine production/development, fair distribution, and increased participation in the vaccination process.

## Methods

A scoping review of the literature was conducted based on Arkesy and O’Malley to identify eligible studies published until November 2021. This protocol consists of six stages: (1) specifying research questions, (2) identifying relevant studies using valid databases, (3) selecting relevant studies, (4) charting data, (5) collating, summarizing, and reporting the results, and (6) voluntarily expert consultation [15].

### Research question

The research questions of this study were: (1) what are the ethical considerations associated with vaccine production? (2) What ethical considerations are associated with vaccine distribution and vaccination? (3) What ethical considerations are associated with specific groups (children and adolescents, the elderly, HCWs, and ethnic and racial groups)?

### Data sources and search strategy

Electronic databases searched were Web of Science, PubMed, Scopus, and SciELO. The search was conducted using combinations of Medical Subject Heading (MeSH) search terms and keywords for Ethics, COVID-19, and vaccines in abstract, keywords, and title fields to retrieve potentially relevant publications. Furthermore, we conducted searches on Google Scholar based on keywords and examined the reference lists of included articles and grey reviews for additional relevant articles. Search syntaxes are accessible in appendices. In addition to a comprehensive search in the databases, we searched gray literature, including theses from ProQuest, conferences from Scopus, and other papers and reports. Furthermore, the search procedure was completed with hand searching (See Additional file 1).

In our study, original articles, reviews, and case studies that were published in any language and examined at least one of the four principles of medical ethics (e.g. autonomy, justice, non-maleficence, and beneficence in the production, distribution, and implementation of vaccinations) were included. Reviewers excluded letters, notes, protocols, and brief/short communications. The eligible studies were entered into Endnote, and duplicates were removed. After removing duplicate records, two independent researchers (H J and M A) independently reviewed the title of the articles and irrelevant ones were removed. In the case of disagreement, the selection was evaluated by a third reviewer (F F-K). Before the final step, the abstract of included studies was reviewed based on the inclusion and exclusion criteria. Finally, the full text of the studies was reviewed, and the final studies were identified to enter the final analysis. Ultimately, 43 papers were deemed adequate for this scoping review.

### Data summary and synthesis

**Fig. 1:**
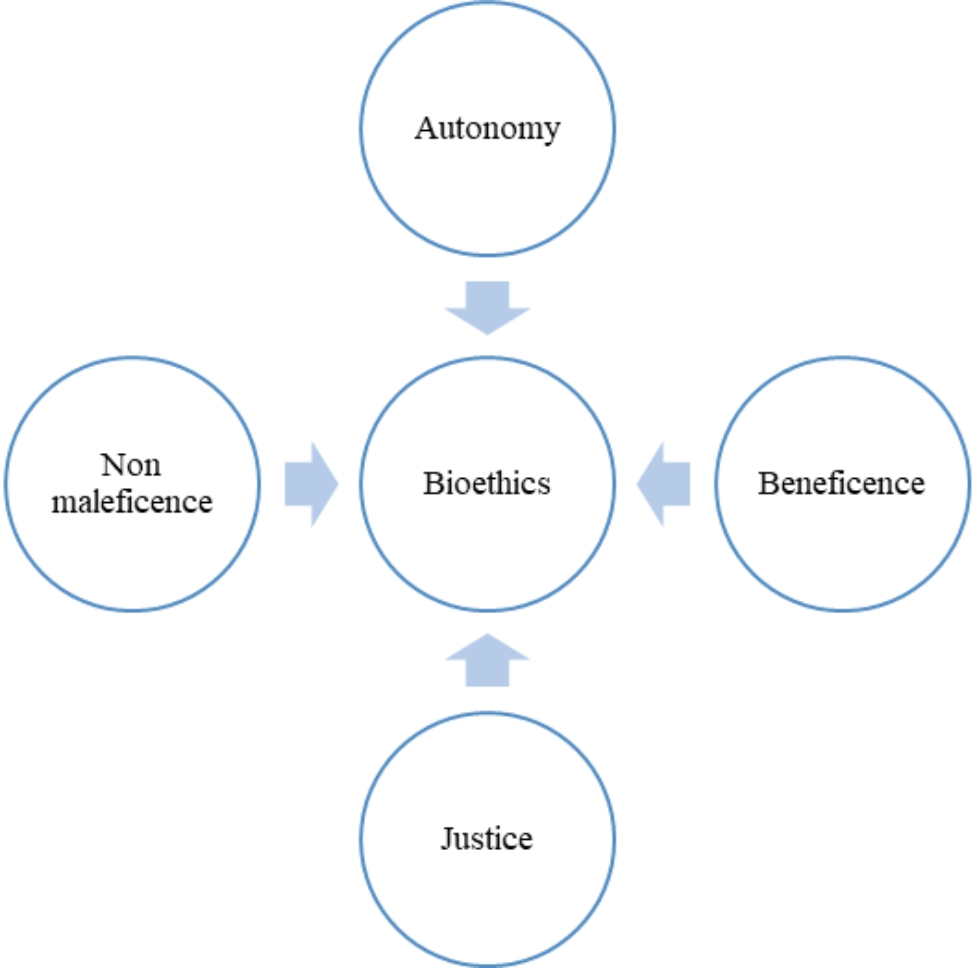
Bioethics framework

We extracted the following data from the included studies: authors’ name, year of publication, study location (s), objective (s), type of article, intervention, ethical principles, and key findings. Data were analyzed using framework analysis based on bioethics principles (justice, beneficence, non-maleficence, and autonomy) in two separate stages of vaccine production as well as distribution and vaccination [8]. Finally, after summarizing and preparing the report, the Triangulation technique, which included researcher surveys, expert opinion polls, and documentation review, was used to validate and enrich the reported data. Figure 1 indicates the main bioethics framework used in this study.

## Results

**Fig. 2:**
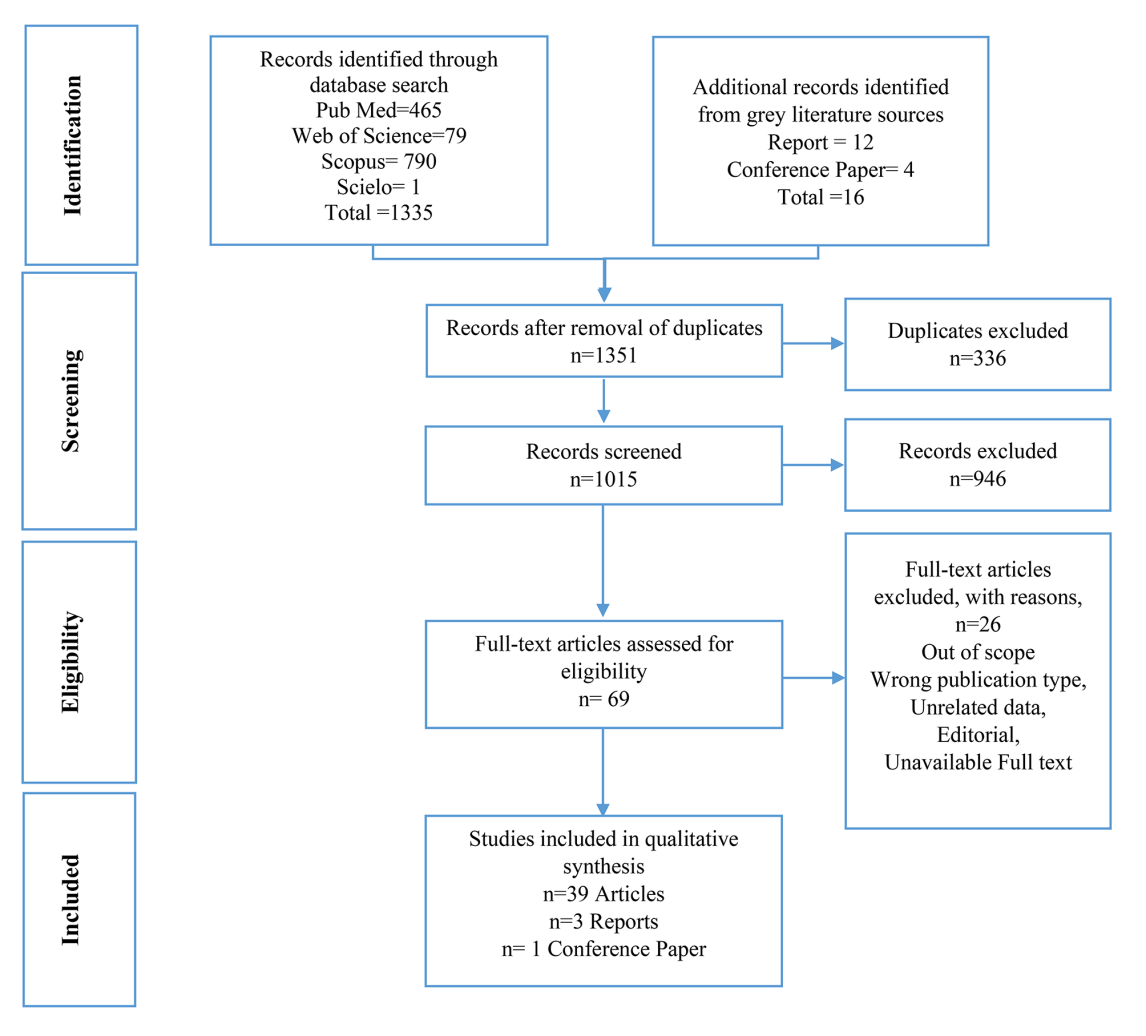
Data acquisition flowchart based on PRISMA

As shown in Fig. 2, search strategies provided 1335 articles, from which 336 duplicates were removed. Based on the title, 946 were excluded. After screening abstracts using inclusion/exclusion criteria, 69 studies remained for further assessment. Finally, a total of 43 articles underwent a full-text review.

The characteristics of included studies are summarized in Table 1. Most of the included studies addressed the principle of justice (n = 33), beneficence (n = 30), non-maleficence (n = 21), and autonomy (n = 19), respectively. In addition, most of the studies addressed the ethical considerations of the process of distribution and vaccination (n = 35), and only a small number addressed the process of vaccine production (n = 14).

**Table 1:** Characteristics of the included studies

No.	Author	Subject	Study type	Scope	Discussed Ethical Issues			
					Autonomy	Non-maleficence	Beneficence	Justice
**1**	**Calina, D. 2021** [4]	Describing the ethical considerations of trial & Intentionally infecting healthy volunteers	Review	Vaccine production	*		*	
**2**	**Dal-Ré, R. 2021** [16]	Challenges of equitable distribution and clinical trials of vaccines in low-income countries	Review	Vaccine production			*	*
**3**	**Daverio, M. 2021** [10]	An overview of ethical and regulatory issues & Vaccine research and the consequences of informed consent	Review	Vaccine production	*	*	*	
**4**	**Greco, D. B. 2021** [12]	Ethical limitations on using placebo and access to Covid-19 vaccines	Review	Vaccine production & distribution		*	*	*
**5**	**John, T. J.** [5]	Covid-19 vaccine trials and ethics	Review	Vaccine production & distribution		*	*	*
**6**	**Kashte, S. 2021** [17]	Challenges and implications of the development of vaccines at present and in the future	Review	Vaccine production			*	*
**7**	**Monrad, J. T. 2020** [18]	Ethical considerations for vaccine trials	Review	Vaccine production		*	*	
**8**	**Vashishtha, V. M. 2021** [2]	Emergency use authorization of Covid-19 vaccines	Review	Vaccine production	*	*	*	*
**9**	**Wibawa, T. 2021** [19]	Ethical considerations of research and development of Covid-19 vaccines	Review	Vaccine production		*	*	*
**10**	**Brusa, M. 2021** [20]	Vaccine policymaking for children in emergencies	Review	Vaccine production & distribution	*	*	*	
**11**	**Dembinski, L. 2021** [21]	Vaccination in children and adolescents	Review	Vaccine distribution		*	*	*
**12**	**Hamidian Jahromi, A. 2021** [22]	Ethical considerations regarding mandatory vaccination for healthcare providers	Review	Vaccine distribution	*	*	*	
**13**	**Hughes, K. 2021** [23]	Ethical considerations and fair prioritization of vaccines in HCWs	Review	Vaccine distribution				*
**14**	**Kates, O. S. 2021** [24]	Ethical considerations of vaccination for transplant center staff and patients	Review	Vaccine distribution	*	*	*	*
**15**	**Bolcato, M. 2021** [13]	Fair distribution of anti-COVID-19 vaccines resources	Review	Vaccine distribution				*
**16**	**Carson, S. L. 2021** [25]	Vaccine Challenges of multiethnic societies	Qualitative	Vaccine distribution	*	*	*	*
**17**	**Adejumo, O. A. 2021** [26]	Global health coverage approach and justice in the vaccine distribution plan	Review	Vaccine distribution				*
**18**	**Binagwaho, A. 2021** [27]	Management of ethical considerations in vaccination	Review	Vaccine production & Distribution		*	*	*
**19**	**Martin-Fumadó, C. 2021** [28]	Ethical considerations of vaccination in health care providers	Review	Vaccine distribution	*		*	
**20**	**Reis-Dennis, S. 2021** [29]	Ethical considerations of vaccines in health care providers	Review	Vaccine distribution				*
**21**	**Savulescu, J. 2021** [30]	Ethical considerations of compulsory vaccination in children	Review	Vaccine distribution	*	*	*	
**22**	**Thorsteinsdottir, B. 2021** [31]	Ethical analysis of prioritizing HCWs for access to the vaccine	Review	Vaccine distribution				*
**23**	**Williamson, L. 2021** [32]	Ethical considerations in the vaccination of children	Review	Vaccine distribution	*		*	
**24**	**Brown, R. C. H. 2021** [33]	Challenges and ethical considerations of immunity passports	Review	Vaccine distribution	*		*	
**25**	**Giubilini, A. 2021** [9]	Ethical considerations of vaccination	Review	Vaccine distribution	*	*	*	*
**26**	**McClung, N. 2021** [34]	Describing the four ethical principles of the Advisory Committee on Immunization Practices (ACIP)	Review	Vaccine distribution			*	*
**27**	**Kumar, V. 2021** [6]	Ethical considerations of vaccine trials	Review	Vaccine production & distribution	*	*	*	*
**28**	**Mirocha, L. 2021** [35]	Ethical considerations of vaccine distribution	Review	Vaccine distribution				*
**29**	**Sharma, P. 2021** [8]	Ethical considerations of vaccination	Review	Vaccine distribution	*	*	*	*
**30**	**Sharun, K. 2021** [36]	Ethical considerations of immunity passports	Review	Vaccine distribution	*			*
**31**	**Voo, T. C. 2021** [37]	Ethical considerations of immunity passports	Review	Vaccine distribution	*		*	*
**32**	**Wang, D. W. L. 2021** [3]	Ethical considerations of mandatory vaccination	Review	Vaccine distribution	*		*	*
**33**	**Woodhead, C. 2021** [38]	Ethical considerations of racial and ethnic group vaccination	Qualitative	Vaccine distribution	*	*	*	*
**34**	**Zhu, X. G. 2021** [39]	Impact of racism and inequality on equitable vaccine distribution	Review	Vaccine distribution	*	*	*	*
**35**	**Emanuel, E. J. 2020** [14]	An ethical framework for global vaccine distribution	Review	Vaccine distribution		*	*	*
**36**	**Gupta, R. 2021** [1]	Ethical issues of vaccine distribution	Review	Vaccine distribution			*	*
**37**	**Liu, Y. 2020** [40]	Providing an ethical framework for fair global vaccine distribution	Review	Vaccine distribution				*
**38**	**McMahon, A. 2021** [41]	Fair global access to vaccines, medicines, and diagnostic equipment and the role of patents	Review	Vaccine production & distribution				*
**39**	**Rhodes, R. 2021** [42]	Ethical considerations of justice in vaccine prioritization	Review	Vaccine distribution				*
**40**	**Hughes, J. A. 2020** [43]	Challenges and implications of new vaccine technologies	Conference article	Vaccine distribution		*		*
**41**	**WHO, 2021** [44]	Ethical considerations of Covid-19 and vaccination	Report	Vaccine distribution				*
**42**	**WHO. 2021** [45]	Review the ethical considerations and dilemmas of decision-making to respond to the epidemic	Report	Vaccine distribution				*
**43**	**WHO, 2020** [46]	Ethical considerations of trials and emergency use of vaccines	Report	Vaccine production		*	*	

Table 2 presents the ethical considerations of the vaccine production process. In our study, the production process consisted of 16 codes and 4 main categories. According to the results of studies, the first ethical imperative is the production and distribution of safe, effective, available, and affordable vaccines, which requires ensuring the use of safe and scientific methodology and technology in vaccine production. Therefore, vaccine production should be performed scientifically and in reputable institutions and comply with the principles, standards, and valid guidelines published worldwide, such as the European Union and the European Medicines Agency (EMA) [4, 8, 10]. Although the vaccine distribution approved by regulatory agencies without completing phase three is not ethically problematic, the risk and benefits of using new technologies must be carefully assessed. They can start using these technologies before finishing clinical trials in order to reduce the harmful and dangerous effects; for the reason that always may be odds of unidentified or not immediate (delayed) hazards that did not be considered in early trial studies [5, 19, 20].

**Table 2:** Ethical considerations in the vaccine production process

Categories	Codes	Semantic units
**Justice**	1- assuring fair access to affordable vaccines for all countries [46].	• Producing affordable vaccines • Considering countries with limited financial capacity • Providing affordable vaccines in the countries participating in the trials
	2- Formulating ethical frameworks to ensure fair and non-exploitative trials in countries with limited resources [18, 46].	• Conducting fair and non-exploitative trials in countries with poor financial resources
	3- Adherence to ethical considerations equally in all countries [12].	• Avoiding double standards for ethics in different countries • Avoiding taking advantage of the legal weaknesses of research ethics in less developed countries
	4- Priority of access for participants in the vaccine trials after approving emergency vaccine use [12, 45].	• Priority of access for participants in the vaccine trial • Clarifying the benefits of the vaccine to participants in trials and making them aware of these benefits.
	5- Comply with the ethical imperatives for issuing patents to manufacturers and issuing mandatory licenses in emergencies [40].	• Limiting the negative impact of patents on the availability and price of Covid-19 vaccines • Revising how patent holders are monitored and controlled • Issuing licenses for a national emergencies (according to ethical imperatives) for the production of vaccines by a third party without the need for licenses of patent owners for fair global access
**Beneficence**	1- Considering the race and demographic characteristics in study designs [16].	• Conducting clinical trials of vaccines at various locations as well as diverse populations at national and global levels
	2- Obligating manufacturers to assure vaccines’ safety, efficacy, and quality [18].	• Considering safety, efficacy, and quality principles for vaccines by manufacturers
	3- Social responsibility and health [19].	• Considering the accountability principle for vaccine manufacturers in countries with poor financial resources
	4- Evaluation of the effectiveness, safety, and risk-benefit of new technologies in vaccine production [18].	• Risk-benefit assessment of new technologies in the production of vaccines • Lack of evidence-based information on the long-term effects of new technologies
**Non-maleficence**	1- Evaluation of study methodology and results by a specialized committee [4].	• Evaluation of methods and results by an independent committee
	2- Formulation of ethical imperatives for using a placebo [12].	• Considering the challenges of using placebos • The right of access to vaccine for the control group after approval • Not depriving participants of the vaccine and appropriate treatment
	3- Transparency in all stages of vaccine development [10, 16, 19, 24, 44].	• Full transparency in vaccine development and production • Dissemination of information by manufacturing companies • Transparency of decision-making processes, including the coordination, development, and production of vaccines • Transparency of information on the scientific method of development, risks, and benefits of vaccines
	4- Respect for human vulnerability and personal integrity [5, 17].	• Protecting vulnerable populations against exploitation
**Autonomy**	1- Consent [6, 10, 16].	• Formulating and designing the informed consent form • Receiving informed consent from participants • Issues related to receiving compensation in the case of any occurrence of complications/side effects from the trials • Awareness of participants about the risks and benefits of trial • Considering issues related to study design in the informed consent form • Considering issues related to the use of placebos in the formulation of the informed consent form
	2- Assuring privacy and confidentiality [10, 32].	• Protecting the privacy of individuals during trials • Protecting the personal information of the participants in trials • Considerations related to access to participants’ medical history
	3- Assuring autonomy and individual responsibility [10, 31].	• Having the right to participate in trials voluntarily Having the right to withdraw from the trial at any stage

In the setting of a clinical trial, vaccine manufacturers should take into consideration inclusion and exclusion criteria such as laboratory and clinical evaluations for including healthy participants in the study. The right of access to vaccine for participants receiving a placebo in a clinical trial should be protected and assured as soon as safety and temporary efficacy are confirmed by the sponsors and health department inspectors, and this right should be clearly stated in the informed consent forms. Furthermore, in the process of inclusion of individuals in clinical trials in the study, any direct or indirect payment for participants should be removed because it may encourage poor people to be exposed to possible risks of these studies solely for economic incentives [2, 6, 10, 12, 18]. Another issue is the availability of a comprehensive treatment center to ensure that trial subjects have access to treatment and care in the case of any serious adverse events related to clinical trial outcomes [19].

Table 3 presents the ethical considerations of the vaccine distribution process and vaccination. The distribution and vaccination process consisted of 12 codes and 4 main Categories. Access to different types of vaccines poses challenges to equitable distribution and prioritization of individuals and communities worldwide. Given that low- and middle-income countries (LMICs) are incapable of producing vaccines and lack sufficient resources to provide vaccines, high-income countries (HICs), the global community, and vaccine companies should provide them with the required logistic support for the implementation of the successful vaccination program.

**Table 3:** Ethical considerations of the vaccine distribution process and vaccination

Categories	Codes	Semantic units
**Justice**	1- Ensuring fair access to vaccines for all countries and communities [13, 34, 46].	• Responsiveness of companies to vaccine fair pricing • Obligating political leaders to provide the vaccine to everyone, especially for high-risk groups • Ensuring global financial resources for vaccine production • Allocating limited resources to the most possible wide range of people fairly (distributive justice) • Prioritization of groups who are at high risk of severe disease and mortality • Unequal allocation is not necessarily unfair
	2- Sharing of benefits [12, 26].	• Considering vaccines as an essential and universal good. • Giving priority to LMICs and other vulnerable countries • Giving priority to minorities or groups who are at higher risk of disease due to social, geographical, or biomedical factors
	3- Respect for cultural diversity and pluralism [12, 26, 40].	• Nationalism can lead to vaccine hoarding and unfair distribution Nationalism may lead to immoral decisions in access to vaccines
	4- Non-discrimination and non-stigmatization [3, 12, 14].	• Setting priorities fairly based on logical parameters tailored to current needs • Using a fair priority model based on limiting harms, priority and benefiting the deprived ones, and equality of people’s value • Transparency in vaccine allocation policies based on rational reasons for decision making
	5- Considering ethical imperatives for immunity passports [32, 35, 36].	• Ethical justification for the implementation of immunity passports, • creating a safe setting for work and travel • The impulsing public will for vaccination • Forecasting mechanisms for issues such as immunity passports’ forgery and ensuring privacy
**Beneficence**	1- Benefit and harm [2, 9, 19].	• Issuance of emergency vaccination authorizations in accordance with WHO guidelines and national medical safety regulations • Assessing the beneficence of vaccines based on evidence-based information • The need to obtain strong evidence on the safety and efficacy of vaccines for compulsory vaccination • Providing evidence-based information on the safety and efficacy of vaccines for vaccination policy • Evaluating the validity of data on the effectiveness, side effects, and safety of vaccines by reputable organizations and specialized assemblies
	2- Monitoring vaccine pricing [26].	• Monitoring the prices of pharmaceutical companies’ products according to government support and subsidies for their research and development
**Non-maleficence**	1- Providing emergency use licenses for vaccines that are proven to be safe and acceptable [8].	• Providing emergency authorization to the vaccines whose safety has been proven. • Dealing with the misuse of issuance of emergency authorizations
	2- Respect for human vulnerability and personal integrity [9].	• Adherence to the harm prevention principle as an ethical imperative for vaccination • Harm prevention is an ethical principle in vaccine studies • The ethical principle of harm prevention entails individual and collective responsibilities • Consideration of harm prevention given the risks and side effects of vaccines
**Autonomy**	1- The conflict between individual autonomy and public benefits [27, 29, 30].	• The conflict between compulsory vaccination and the free will or individual autonomy concept • Adherence to the three necessary criteria for the ethical justification of compulsory vaccination, including the threat to the disease for public health, the expected positive beneficence, and the proportionate coercion. • Implementation of a mandatory vaccination policy only if it is possible to prevent significant risks of disease and mortality or significant and clear public health benefits.
	2- Autonomy and individual responsibility [25].	• Considering the factors influencing decision-making processes to increase acceptability, accessibility, and justice • Clarifying information about the safety and effectiveness of vaccines to address doubts about informed decision making
	3- Human dignity and human rights [32, 35].	• Acknowledging the rights and interests of participants • Identifying people who are immune to the disease • Providing the requirements for immune individuals to return to work or/and to do their activities • Adherence to ethical autonomy imperatives in immunity passports • Protecting participants through a balance between potential risks and benefits

In vaccine distribution, two main considerations need to be taken into account: (1) the ability of vaccine development and trial and (2) purchasing power. These two principles should be considered in the justice and beneficence of vaccine distribution [12, 13, 26, 35, 40]. According to the Advisory Committee, the four principles, including maximizing benefits and minimizing harm, promoting justice, mitigating health inequalities, and promoting transparency, should be taken into account for the allocation of the COVID-19 vaccine [6, 34].

Lack of trust in the beneficence and safety of the COVID-19 vaccine stemming from distrust in scientific research, insufficient evidence and information and exposure to invalid information about the vaccine, fear of politicization, and misuse of vaccine by the pharmaceutical industry can affect the public acceptance of COVID 19 vaccine. A possible unintended effect of compulsory vaccination can reduce people’s desire to get the vaccine. Therefore, a valid information system to provide information on vaccines to the community is required for their informed decision [25, 28]. The allocation and distribution of the COVID-19 vaccine should maximize the benefits of vaccination for both individual recipients and the general population [6, 34]. Moreover, if everyone has access to the vaccine, there will be a considerable ethical justification for enforcing vaccination passports, as it creates a safer environment for work, travel, and other routine activities. It is unethical to prevent unvaccinated people upon arriving in a country. Therefore, immunity passports should allow the holder to travel freely within the country, and unvaccinated travelers should be subject to restrictions upon arrival in a country and should undergo quarantine [33, 36, 37].

**Fig. 3:**
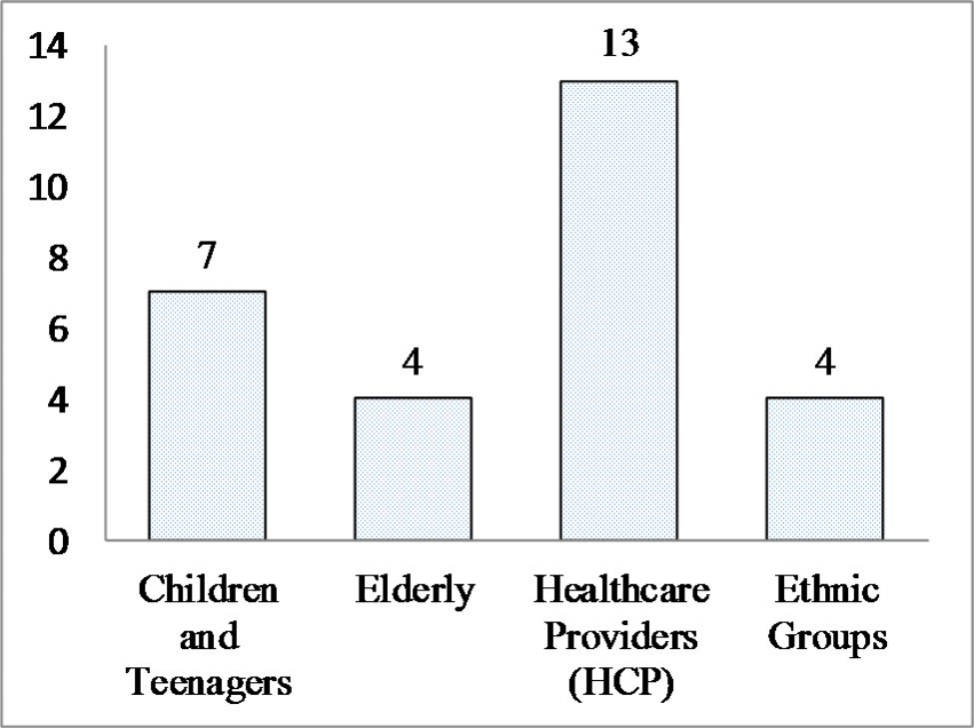
Frequency of articles published in each group

Table 4 presents ethical considerations for specific groups. According to the results, out of 43 studies, 13 examined ethical considerations related to HCWs, 7 examined children and adolescents, 4 examined the elderly, and 4 examined ethnic and racial groups. Most of the included studies examined ethical considerations related to healthcare workers (Fig. 3).

**Table 4:** Ethical considerations for specific groups based on each of the included studies

	Specific Groups			
	Children and Teenagers	Elderly	Healthcare Providers (HCP)	Ethnic Groups
**John, T. J.** [5]		*	*	
**Brusa, M.** [20]	*			
**Dembinski, L.** [21]	*	*		
**Hamidian Jahromi, A.** [22]			*	
**Hughes, K.** [23]			*	
**Kates, O. S.** [24]			*	
**Carson, S. L.** [25]				*
**Martin-Fumado, C.** [28]			*	
**Reis-Dennis, S.** [29]			*	
**Savulescu, J.** [30]	*			
**Thorsteinsdottir, B.** [31]	*		*	
**Williamson, L.** [32]	*			
**Giubilini, A.** [9]			*	
**McClung, N.** [34]			*	
**Kumar, V.** [6]				
**Mirocha, L.** [35]			*	
**Woodhead, C.** [38]				*
**Zhu, X. G.** [39]				*
**Gupta, R.** [1]	*	*	*	
**Rhodes, R.** [42]			*	
**WHO** [44]	*	*	*	*

Table 5 presents the ethical considerations of specific groups. Given that most medicines used for children have never independently undergone specific clinical trials, it would be inappropriate to provide strict standards for the approval and authorization of COVID-19 vaccines. However, parents and adult children need to be informed about the side effects of vaccines. Moreover, one of the ethical concerns of the allocation of vaccines is vaccinations for children and adolescents who are less prone to severe COVID-19 but still consume vaccine resources that can be used more efficiently for immunization of high-risk groups. This is especially important in developing and underdeveloped countries with limited resources. Considering that children volunteering for a new vaccine that is not a serious threat to them, and mandatory vaccination for them depends on detailed information about the nature of the disease and its severity and prevalence, we must consider the expected usefulness to society and the child ethically [2, 21, 30, 32].

**Table 5:** Ethical considerations related to specific groups

Specific groups	Codes	Semantic units
**HCWs**	1- Prioritizing front-line HCWs due to their higher odds of contagion exposure [28, 30, 41, 43].	• Giving priority to HCWs due to constant exposure • Respecting the right to have a safe work environment • The importance of the ability to care for and protect patients • Maintaining the stability and resilience of the health care system in emergencies
	2- Conflict of ethical principles of autonomy and beneficence with compulsory vaccination among HCWs [16, 21, 42].	• Consideration of the conflict between the ethical principles of beneficence and autonomy with compulsory vaccination of health care workers
	3- Concerns of HCWs about the safety and effectiveness of vaccines [21].	• HCWs doubt the safety and effectiveness of vaccines • HCWs worry about the severity of the disease • HCWs have concerns about Covid-19 and vaccination like other people in society
	4- The unwillingness of HCWs to be vaccinated despite their responsibility to implement the vaccination strategy and reassure the community [27, 42].	• Responsibility of HCWs as the main element in implementing public vaccination strategies • HCWs’ responsibility to reassure the community • The reluctance of HCWs due to uncertainty about the safety of vaccines • The effect of race on vaccine hesitancy and primarily unequal access among HCWs
	5- Prioritizing vaccination based on balancing profit and loss/ benefits and risks (balance of principles of beneficence and non-maleficence) [27, 42].	• Consideration of maximizing vaccination benefits and minimizing risks to prioritize HCWs • Vaccination is more useful for HCWs because of the greater risk of infection • High risk of infection in HCWs due to their constant exposure
**Children and adolescents**	1- Acquiring informed consent tailored to children and adolescents [9, 31].	• Considering the ethical considerations of autonomy, informed consent, confidentiality, and protection of individuals for vaccination of children like other groups • Acquiring informed consent from children for vaccination like any other medical procedure
	2- Evaluation and acceleration of vaccine immunization trials vaccine in children and adolescents [20].	• Accelerating immunogenicity trials of Covid-19 vaccine in children and adolescents due to lack of reliable, evidence-based information on their long-term effects
	3- Consideration of the expected beneficence for the community and the child during the children and adolescents vaccination process [29].	• Consideration of the expected beneficence for the community and the child during the children and adolescents vaccination process • Implementation of compulsory vaccination policies for children can be justified when it has the expected positive benefits for both society and children and adolescents.
	4- Considering the ethical principles of autonomy, non-maleficence, and trust in the vaccination of children as well as adolescents [9, 31].	• Impossibility of acquiring informed consent from the children without the parent’s permission • The negative impact of parental pressure to vaccinate children and adolescents on their autonomy and informed consent
	5- Lack of ethical justification for the vaccination of children as a requirement to attend school [43].	• Lack of evidence-based information on the safety and efficacy of Covid-19 vaccines for children • Vaccination requirement for children to attend school is not ethical
**Elderly**	1- Giving priority to the elderly [4, 20].	• Giving priority to the elderly for injecting emergency licensed vaccines • Giving priority to the elderly with comorbidities
**Ethnic and racial minorities**	1- Identifying the conditions and social harms of racial and ethnic minority communities to remove barriers to vaccination [24, 38].	• Limited access of ethnic and racial minorities to health care and services due to social factors • Identifying the conditions and social harms of ethnic and racial minorities
	2- Considering institutional and structural discrimination as a factor leading to vaccine hesitancy among ethnic and racial groups [22, 24, 37, 38].	• Suspicion, fear, and hesitancy about the pressure to be vaccinated among minorities • Racial injustices in vaccine development and trial • Minorities’ religious and moral concerns • Hesitancy about the legitimacy and restrictions on access to vaccine information • The rampancy of distrust and uncertainty among minorities due to previous institutional and structural discrimination
	3- The necessity of reducing health inequalities in the distribution of vaccines [38].	• Opportunity-oriented approach to the distribution of the Covid-19 vaccine as an opportunity to address the health inequalities of ethnic and racial minorities • The effect of the socio-economic status of ethnic and racial minorities on the fair distribution of vaccines • Coping with the unfair distribution of vaccines among minorities (removing barriers to equal distribution of vaccines among ethnic and racial minorities)

Given the close relationship between HCWs and the patient and the importance of healthcare system resilience and assuring continuity of health service provision in emergencies, there are strong and valid ethical justifications to give priority access to HCWs for COVID-19 vaccination [5, 29, 31, 41]. However, mandatory vaccination for HCWs conflicts with the principles of autonomy and beneficence because they may prefer a known disease to unknown side effects of vaccines. Further, providing false and non-transparent information contributes to their vaccine hesitancy [22]. Considerations of the principles of beneficence and non-maleficence to the patient necessitate the vaccination of HCWs, which justifies some intrusion on staff autonomy [24]. Additionally, in the condition that the vaccination rate was required for ensuring disease control and it was not achieved through voluntary vaccination, compulsory vaccination of health workers could be justified ethically [28]. Hospitals that lack sufficient resources to vaccinate entire their staff should morally prioritize their staff and take into account considerations such as the possibility of infection and transmitting contagion as well as the rate of absenteeism [29, 34]. The codes categorized in Table 5 are in accordance with the “Respect for cultural diversity and pluralism”, “Persons without the capacity to consent” and “Protecting future generations” principles in the UNESCO Universal Declaration on Bioethics and Human Rights [47].

**Fig. 4:**
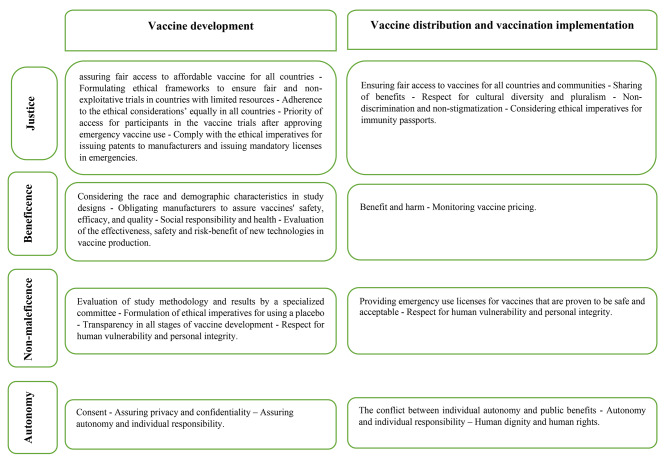
Matrix of ethical considerations of the vaccine development process and vaccination

After the synthesis of the findings, we developed the final framework of ethical considerations related to emergency vaccination (pandemics and epidemics) (Fig. 4).

## Discussion

Our study aimed to integrate the results of different studies on the COVID-19 vaccine to clarify and classify the ethical considerations into two categories: (1) Vaccine development, and (2) Distribution and implementation of vaccination. The results showed that considerations related to the four principles of bioethics, including justice, non-maleficence, autonomy, and beneficence, should be considered at each stage of COVID-19 vaccine development and in the process of distribution and vaccination.

In concern of the justice principle, equitable vaccine distribution may be very difficult or even impossible due to the limited production of approved COVID-19 vaccines and the high demand of countries and nationalism. The capacity and capability of the health systems in different countries, especially LMICs, in comparison with HICs that have the ability to produce and distribute vaccines widely, poses fundamental challenges to the principle of justice. To establish equality and equitable distribution, the COVAX initiative was established to provide fair access for all countries and prioritize vulnerable groups such as the elderly and HCWs [26].

Despite the global epidemic, granting patents to manufacturers could impede the fair distribution of the vaccine worldwide and monopolize the price of the vaccine. Therefore, global and pandemic emergencies can be a factor in revising the degree of control of patent holders and allowing third parties to produce based on some emergency licenses without the need for a license from a patented manufacturer. According to the “Social responsibility and health” principle of the UNESCO Universal Declaration [47], the main purpose of governments is health promotion and social development for their people. Consequently, they need to neutralize patents’ negative effects in cases such as pandemics. To cope with the problem, COVAX, a platform to support the research, development, and manufacturing of a wide range of COVID-19 vaccine candidates and negotiate their pricing, has been established to accelerate the development, allocation, and equitable access to COVID-19 vaccines through a global collaboration between governments, intergovernmental organizations, and private sectors [48].

Moreover, vaccine developers have taken into account principles of corporate social responsibility (CSR), as a ‘win–win’ strategic management tool for accomplishing sustainable development [49] during COVID-19. A study suggested that corporate social sustainability in addition to reducing the risk of litigation and financial difficulties can protect companies from boycotts and other actions by different stakeholders [50]. CSR also has some ethical practices such as equity, justice, and social care without benefiting the company’s economic and financial situation [51] which are morally obligatory.

It has been shown that the participation of pharmaceutical companies through COVAX is significantly low [52]. This can be due to sidestepping of COVAX by rich and developed countries, as they have made bilateral agreements directly and often with multiple vaccine manufacturers, and have bought most of the doses now and for years to come [53]. A strategy that can lead to an increase in the global supply of COVID-19 vaccines worldwide is making manufacturing know-how and formulas openly and freely available by removing patent protections temporarily [53]. Based on the principles of “Sharing of benefits”, “Respect for cultural diversity and pluralism” and “Non-discrimination and non-stigmatization”, it is necessary to share benefits from any scientific research and its applications with the international community, especially with LMICs [47]. Another strategy is the waiver of intellectual property (IP) protections on Covid-19 vaccines [53]. A temporary patent waiver can save more lives of individuals, especially those living in LMICs. Undoubtedly, IP law can exacerbate both global and domestic inequalities, which in turn, is a violation of states’ human rights obligations to respect, protect and fulfill the right to health. Also, the commodification of essential medicines, such as vaccines, pushes poorer countries into extreme debt. The best way to address the inequalities and injustices is through a decolonized approach to human rights [54]. Patent right is accepted by all parties, while, establishing “prime the pump” mechanisms in market-based economies to provide incentives to the pharmaceutical industry to research vaccine development needs a more scrutinized process in developed countries. Implementing programs such as ‘Operation Warp Speed’ that aimed to provide substantial quantities of safe and effective vaccines for Americans based on government financial support requires localization studies and adjustments in developing countries [55].

Ensuring fair access and distribution of vaccines both among countries and within communities based on high-risk and at-risk groups are ethical imperatives of the distribution and vaccination process requiring special attention and planning. The challenge of access to vaccination is the most important problem in the solving of COVID-19 difficulties. All countries do not have equal access to the vaccine, and there is a hegemony in access to vaccines, as third-world countries lack the power to procure the vaccine [56]. vaccine nationalism hinders the world’s poorest countries to have access to vaccines and can be a threatening factor to vaccine distribution [47, 57]. A common hindrance is the lack of enforcement mechanisms for human rights or the lack of funding with WHO [56]. This may not be possible for all people to have access to the vaccine during a pandemic like COVID-19 when due to vaccine nationalism, rich countries purchase the majority of vaccines. Hence vaccine nationalism can prevent the vaccine from reaching vulnerable people in poor countries [57]. Enforceable frameworks for vaccine development and distribution are needed in order to encourage the international sharing of vaccines (6R) [57]. The human right of equal access to the vaccine can be achieved through the collaborative help of WHO and all governments across the world. Further, under Article 19 of the WHO Constitution, this organization should adopt a convention for access to vaccines during a pandemic [56]. Furthermore, prioritization processes must be rational, need-based, and fairly given communities’ social, geographical, and biomedical factors.

According to the principle of beneficence, it is necessary to have a comprehensive approach to the principles of beneficence and non-maleficence. In this way, public benefits overweight public harms. Therefore, the principle of beneficence deserves particular attention both in clinical trials and in vaccinations. To do so, researchers should, both at the vaccine development, and vaccination stage, take measures that the accumulative benefit of public health interventions outweighs the side effects and have a considerable role in preventing and controlling the COVID-19 outbreak. Further, according to the principle of non-maleficence, researchers should minimize the possibility of adverse effects of the COVID-19 vaccine on participants through various ways such as the use of scientific and valid methods, the use of accepted international standards, and monitoring the process of development of new technologies in vaccine development. This is implied in the “human vulnerability” article of the UNESCO Universal Declaration on Bioethics and Human Rights that Individuals and groups of special vulnerability should be protected and taken into account [47].

Our results showed that in the process of vaccine production and development for Risk assessment and management, specialized committees should address the methodology, safety, and efficacy of vaccines for evaluation, as well as access and transparency of information about vaccine production, efficacy, and side effects should be assured. Ensuring the safety and efficacy of vaccines is also a necessary precondition for mandatory vaccination, requiring access to information based on credible evidence that must be available publicly. Addressing bioethical issues such as transparency seems necessary in vaccine safety and effectiveness for appropriate sharing of knowledge and avoiding conflicts of interest, in addition, it can increase the desire of people to receive vaccines by resolving vaccine hesitancy and uncertainty. Therefore, neutral third parties must evaluate safety and effectiveness outcomes at the national and international levels.

According to the principle of autonomy, participants need to sign an informed consent during trials. The content of these forms should include various ethical considerations such as side effects and consequences of trial, how to compensate for damages, and the use of placebos in order to increase participants’ informed decision-making power. Autonomy, priority, and protection against the exploitation of individuals and contributing countries need to be factored in. furthermore, it is necessary that governments, public and private institutions as well as professionals related to transnational activities act according to international laws, standards, and ethical principles [47]. In the process of distribution and vaccination, while paying attention to the autonomy of individuals, the priority of the principle of beneficence over the principle of autonomy in vaccination should be considered due to the existence of external consequences. Moreover, in case of a pandemic threat at a national level, the principle of beneficence can affect the principle of autonomy. Reliable information about vaccines’ immunogenicity should be available to vaccinate children and adolescents. Given the role of parents in deciding about accepting the vaccine, vaccine trials should guarantee the ethical principles of autonomy and non-maleficence for both children and adolescents after their informed consent.

Because HCWs are more vulnerable and at a higher risk of being infected, they should be a top priority in accessing the COVID-19 vaccine. HCWs also have concerns about the safety and effectiveness of vaccines that should be considered. Furthermore, given that their mandatory vaccination conflicts with the ethical principles of autonomy and beneficence, there must be sufficient moral justification in this regard. The COVID-19 pandemic showed that HCWs are the most important pillar of health care systems in the face of pandemics, and maintaining their safety and health leads to leads to ensuring continuity of health care provision. Therefore, governments should formulate programs to support them in increasing the resilience of health systems and managing future pandemics.

Ethnic and racial minorities are hesitant to get the vaccine owing to institutional and structural discrimination such as religious concerns, institutionalized health inequalities, inequalities in access to information and racial injustices in the vaccine production and trial process, fears and suspicions of the exercise of power, the compulsion to be vaccinated, the legitimacy and access to vaccine-related messages and information. In order to reduce doubts and uncertainty and reduce health inequalities and fair distribution of vaccines among them, these barriers should be removed.

Given the urgent need of communities in emergencies for safe treatments and vaccines, the role of ethics committees in adhering to ethical principles and frameworks in vaccine clinical trials is highly important than ever. Given the responsibilities for protecting the rights and health of participants, continuously monitoring and evaluating trial protocols and guidelines, and ultimately monitoring the proper implementation of these principles, ethics committees should be more prepared for similar cases and provide ethical frameworks for the development, distribution, and appropriate vaccination processes of pandemics and epidemics for faster decision-making. International health organizations should also play a more active role in regional epidemics and pandemics and anticipate and prepare policymaking frameworks in cooperation with national governments and the commitment of political leaders. In similar cases and regional epidemics, the implementation of ethical considerations related to the development, distribution, and vaccination of countries should be recognized as an obligation to ensure the realization of the principle of beneficence, justice, and non-maleficence to the safety of communities. Moreover, in line with international cooperation, states should build solidarity, and encourage the free dissemination of information and access to technologies [47].

In developing and formulating these frameworks, countries’ cultural and moral differences should be taken into account. Therefore, we recommend that countries develop and formulate ethical guidelines and frameworks that are consistent with their own countries’ ethical imperatives during pandemics and emergencies. Moreover, our suggestion is that future studies formulate executive ethics guidelines based on the ethical principles presented in this study for future pandemics.

The examination of the quadruple ethical principles associated with COVID-19 vaccination shows that policymakers in this regard face numerous ethical dilemmas stemming from a conflict between these four principles. Therefore, it seems that the realization of these principles simultaneously is very difficult and impossible, and policymakers should prioritize some principles over others. Dividing ethical principles into three levels: individual, national, and international can solve some of these ethical dilemmas. At the individual level, the ethical principles of non-maleficence and autonomy are made a priority, while at the national level, the ethical principles of justice and beneficence are prioritized over the principle of autonomy. At the national level, the dominant political ideology in society is very decisive. Importantly, at this level, issues of nationalism should not affect the fair distribution of vaccines among vulnerable groups. Finally, given the international nature of the pandemic and the need for coordinated and integrated international action to control the pandemic, the principle of beneficence is a priority. Therefore, the final criterion for deciding should be based on a comparison between the public benefits and the public risks of vaccination, although this criterion conflicts with the principle of autonomy.

### Limitations

Due to the persistence of the COVID-19 pandemic, there are still unknown numbers of studies in progress or in print that were not available at the time of our search. However, we tried to minimize this limitation using the triangulation technique.

## Conclusion

Given the externalities of pandemics (transmissibility of disease to others) and the collective and social benefits and harms, it is impossible to adhere to all four principles of medical ethics simultaneously and completely. This may confront individuals and policymakers with many ethical dilemmas. It seems that decision-making based on the balance between social benefit and social harm is a better criterion in this regard, and the final decision should be made based on maximizing the public benefits and minimizing the public harm. This principle can be used as a general guiding principle for deciding when ethical dilemmas arise. As a whole, it can be concluded that the principle of beneficence is the predominant principle of bioethics concerning COVID-19 vaccination and can affect other principles (e.g. justice, autonomy, and non-maleficence). In regional epidemics and similar pandemics, ethics committees and international health-related organizations need to play a more active role and consider more comprehensive, efficient, and effective ethical frameworks for responding to emergencies.

## Data Availability

All data generated or analyzed during this study are included in this published article.

## Abbreviations

**HCWs**: Healthcare workers
**HCTs**: Human Challenge Trials
**LMICs**: Low- and middle-income countries
**HICs**: High-income countries
